# Towards a catalogue of biodiversity databases: An ontological case study

**DOI:** 10.3897/BDJ.8.e32765

**Published:** 2020-03-27

**Authors:** Jarrett Blair, Rodger Gwiazdowski, Andrew Borrelli, Michelle Hotchkiss, Candace Park, Gleannan Perrett, Robert Hanner

**Affiliations:** 1 University of Guelph, Guelph, Canada University of Guelph Guelph Canada; 2 University of Massachusetts Amherst, Amherst, MA, United States of America University of Massachusetts Amherst Amherst, MA United States of America

**Keywords:** Ontology, Database, Databases, Database of Databases, Metadata, Biodiversity, Indexing, Information Resource Discover

## Abstract

Biodiversity informatics depends on digital access to credible information about species. Many online resources host species’ data, but the lack of categorisation for these resources inhibits the growth of this entire field. To explore possible solutions, we examined the (now retired) Biodiversity Information Projects of the World (BIPW) dataset created by the Biodiversity Information Standards (TDWG); this project, which ran from 2007-2015 (officially removed from the TDWG website in 2018) was an attempt at organising the Web's biodiversity databases into an indexed list. To do this, we applied a simple classification scheme to score databases within BIPW based on nine data categories, to characterise trends and current compositions of this biodiversity e-infrastructure. Primarily, we found that of 600 databases investigated from BIPW, only 315 (~53%) were accessible at the time of this writing, underscoring the precarious nature of the biodiversity information landscape. Many of these databases are still available, but suffer accessibility issues such as link rot, thus putting the information they contain in danger of being lost. We propose that a community-driven database of biodiversity databases with an accompanying ontology could facilitate efficient discovery of relevant biodiversity databases and support smaller databases – which have the greatest risk of being lost.

## Introduction

A wealth of biodiversity data is available online and dynamically indexing these often disparate resources will be pivotal for all the biodiversity sciences (Ball-Damerow et al. 2017, Peterson et al. 2010). The inherent heterogeneity of biodiversity information found within and across databases can make it difficult to access and assemble for analyses. (Parr et al. 2012). Even large, well-established databases often contain outdated or unreliable data. For any one species, data are often scattered across multiple databases making the discovery and curation of a dataset from disjointed data a significant barrier for any study (Hobern et al. 2014, Peterson et al. 2010). Additionally, pseudo-replication within and between databases is compounded by a lack of metadata (Hardisty et al. 2013, Peterson et al. 2010); this is a particular issue with occurrence data - the major data type for informing policy developments (Hobern et al. 2014). Generally, within databases, the lack of standardised information about data categories and their metadata (contextual information about primary data describing when, where and how data points were collected) impedes both automated or manual bioinformatic analysis (Peterson et al. 2010, Michener 2006).

**Figure 1.**
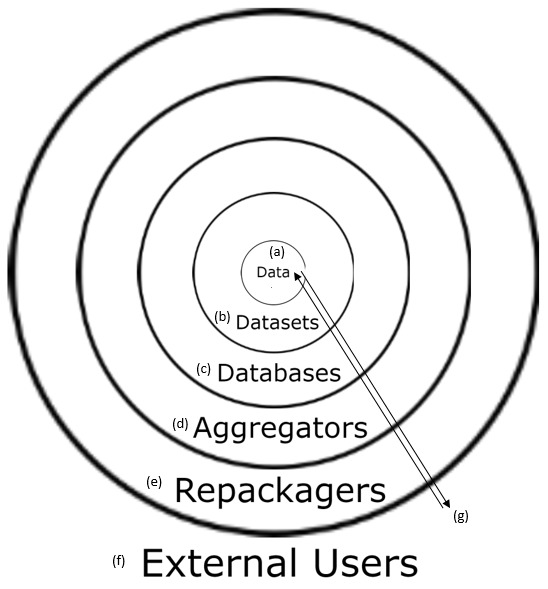
(a) Data and metadata make up (b) datasets. Multiple datasets in one location form a (c) database. (d) Aggregators compile data from many databases and (e) repackagers transform data in a way that makes it more accessible for all audiences (i.e. lay and professional). (f) External users (e.g. scientists, industries, government agencies etc.) access raw data by (g) going through any or all of these data sharing portals. This figure was adapted from Andy Bentley's presentation at the 2017 inaugural iDigBio conference (https://www.idigbio.org/wiki/images/4/4e/Natural_History_data_pipelines_-_Bentley.pdf).

Data linked with its metadata are the foundation of all databases because it permits rapid quality control for analyses, at all scales (Fegraus et al. 2005, Hampton et al. 2013). To standardise searches for data & metadata, data-standards initiatives such as the Darwin Core establish basic glossaries of terms (~200 in Darwin Core) (Wieczorek et al. 2012); whereas other standards, such as Access to Biological Collections Data (ABCD), use a finer grain of terms (~1200 in ABCD) in a hierarchical scheme reflecting semantic relationships between real world objects or concepts (Holetschek et al. 2012, Walls et al. 2014). Further, other standards by the Biological Collection Ontology (BCO) use terms within a proper ontology following a subject-predicate-object format (Walls et al. 2014). Data standards help circumvent the myriad complications in managing data from diverse sources (Kolb et al. 2013) and only with data standards are the analyses of large-scale datasets possible (Wieczorek et al. 2012). As a result of data standards, large data-aggregators, such as the Global Biodiversity Information Facility (GBIF) which hosts over 1 billion species occurrence records, can now make datasets from multiple providers accessible via a single interface (Global Biodiversity Information Facility 2018, Holetschek et al. 2012) (Fig. 1).

Despite the benefits of data standards and data-aggregators, a fundamental issue for biodiversity data is simply finding databases which host specific data. Like their data, biodiversity databases are diverse, with some having a narrow or local focus and others, like GenBank, with broad content. Search engines like Google are ineffective because many biodiversity database websites are not optimised for search engine discoverability and generic search engines cannot sort databases by data category. Additionally, while aggregators excel at compiling datasets, they often lack important intricacies relative to data categories or data structure, where a specialised database presents a clearer delivery of the information it hosts.

A promising solution to develop a classification scheme for categorising biodiversity databases is to mirror practices from data standards. In doing this, specific types of data hosted within databases will need to be explicitly identified by standard categories. Due to the diversity of data types in biodiversity databases, large categories (e.g. taxonomic data, geospatial data etc.) could be divided into sub categories (e.g. taxonomic resolution, taxonomic groups, geographic resolution etc.). The large categories could operate on a binary system (e.g. Taxonomic data = Yes), while sub categories would primarily operate on searchable keywords (e.g. Taxonomic groups = Buprestidae, Coccinellidae). Like existing data standards and ontologies, each category could have an associated definition, examples and commentary.

Currently, biodiversity databases are typically indexed within simple lists, devoid of database metadata. To explore a practical solution for categorising diverse and complex biodiversity databases, this case study accessed and evaluated an extensive list of databases provided in 2007 by Biodiversity Information Standards - also known as the Taxonomic Databases Working Group (TDWG) (Biodiversity Information Standards 2008). The TDWG list is one of the earliest attempts at curating a list of biodiversity databases and even included some metadata about the databases such as author’s names, geographic scope and taxonomic scope. At the time of this study, the TDWG website has been redesigned and the list no longer exists on the website. However, we are still able to see how the website looked using the Internet Archive (i.e. Wayback Machine) and the list’s data are publicly available on GitHub (Belbin et al. 2007, Biodiversity Information Standards 2008). Using these resources, databases from the TDWG list were scored based on the presence or absence of nine data categories, to address the following three questions: 1) what type of information can be found in this biodiversity e-infrastructure; 2) what is the morphological nature (i.e. size and shape) of the information's whole and individual components and; 3) where can each type of information be accessed?

## Methods

We examined 600 databases (accessible, from a total of 685) collected from the Biodiversity Information Projects of the World (BIPW) website by TDWG (Belbin et al. 2007, Biodiversity Information Standards 2008). To do this, we first searched the database titles via Google’s search engine (the method we expect most researchers would first use) and omitted websites by any of four criteria: 1) the link for the database provided by Google was broken; 2) if the database had an ambiguous name and search results (i.e. Field Guide to Insects); 3) the database appeared to have changed names; 4) the search only returned a journal article and/or no data was actually provided by the website. Accessible databases were then examined by three characteristics: 1) identify its current URL; 2) determine the year it was last active; and 3) Score its data contents based on nine categories (detailed in Table 1, below).

Categories were created subjectively from common data types found across databases. Each database was scored using a binary system of 0 or 1 for each category (Table 1). For example, if a database only contained DNA sequence, geospatial and temporal data, it would be scored as a 1 for each of those three categories (totalling 3) and given a 0 for every other category. It is also possible for a database to be scored as a 0 for every category. Some of the “databases” included on the BIPW were not actually databases, but instead were informational web pages about specific projects or organisations. These “databases” did not contain any data and were thus given a 0 for every category. Transition to a binary scoring scheme required an assessment on appropriate levels of exclusivity for each category. Therefore, two people independently scored each database and scoring conflicts were resolved through discussion. The authors readily acknowledge the limitations of this approach and propose this to be an appropriate measure to develop a proof-of-concept. After scoring, we observationally graphed the results to illustrate this sample of a biodiversity e-infrastructure. The figures depict the proportion of omitted vs. accepted databases (based on our criteria), the proportion of databases that were last active in specified years and the proportion of databases providing information within each of the categories defined herein (Table 1). Databases that contained no data for all categories were removed from the list.

## Results

**Figure 2.**
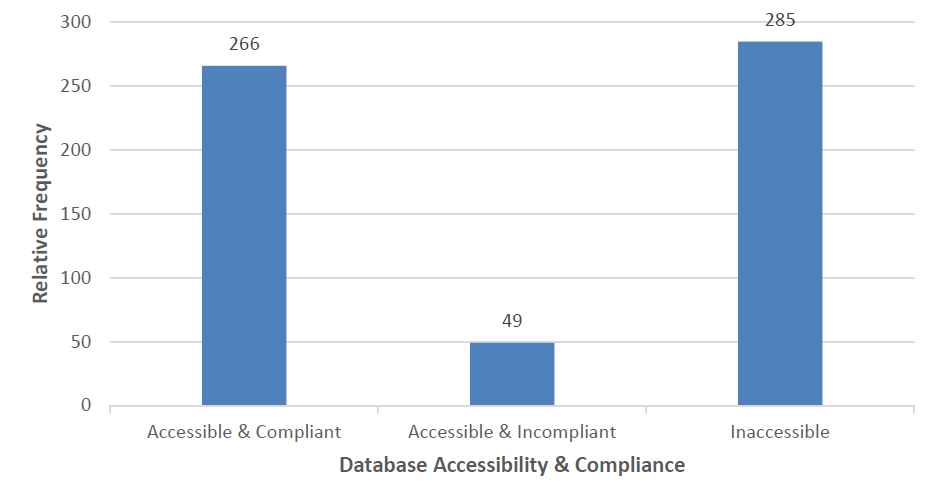
Depiction of the relative amounts between accessible, with or without our categorical criteria and inaccessible databases (n=600) from the investigated TDWG list.

Out of the 600 databases, we found 315 (52.5%) were accessible and 285 (47.5%) were omitted (Fig. 2). Additionally, 49 of the 315 databases contained information which did not fit any of our data categories. These databases scored 0 for each category, reducing the effective list to 266 databases (43.3%) of the 600.

**Figure 3.**
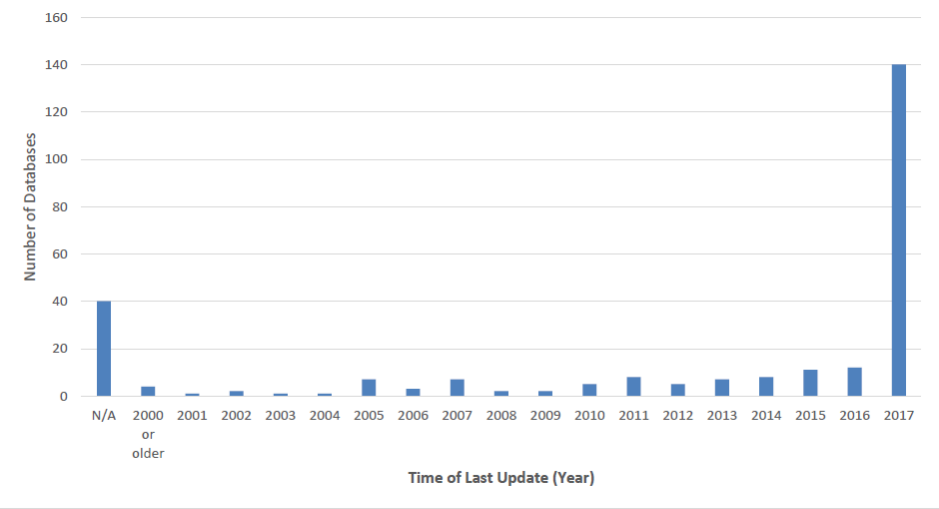
Most recent activity of all 266 databases that were accessible and compliant, based on information provided within each database. Categorisation of activity levels was based on yearly increments unless activity information was unavailable online (N/A).

Of these 266 databases, 139 (52.3%) were actively updated at the time of our analysis (2017), 41 databases (15.4%) did not indicate last-updated information (Fig. 3) and the remaining 86 (32.3%) databases had no updates since 2017.

**Figure 4.**
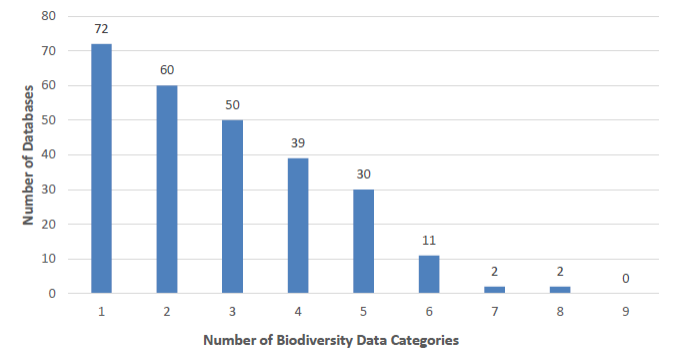
Depiction of how frequently a database simultaneously hosted information complying with any number of data categories, according to our definitions, throughout the accessible and compliant databases (n=266).

The biodiversity databases (n=266) that were accessible and complied with at least one of our criteria, were organised into bins based on the number of categories that had relevant information. Notably, 21.7% of the viable databases complied with only one of the given criteria (Fig. 4). Databases with nine simultaneously hosted categories had the lowest frequency, 0%. Databases that contained no data for all categories were removed from the list. There appears to be a negative relationship between number of categories a database provides and the number of databases, where the number of databases appears to decrease with increased breadth of information (Fig. 4). When looking at the average score of all of the 266 databases evaluated, the value was 2.80, indicating that, for each database, it met the criteria for almost three out of the nine biodiversity data categories.

**Figure 5.**
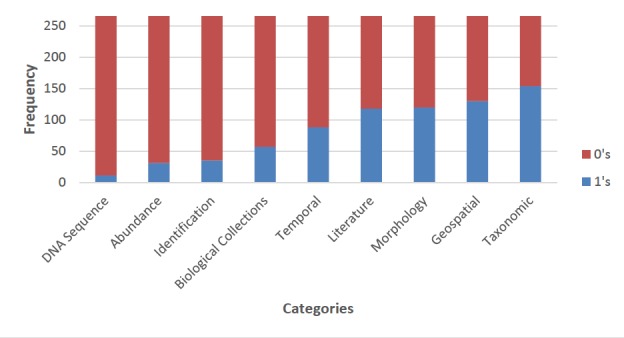
Depiction of the accessible biodiversity databases (n=266) characterised by binary scoring values (0 and 1), based on compliance of information for each criterion analysed.

All categories, aside from taxonomy, had a majority of databases pertaining a score of 0, indicating that most of the biodiversity databases did not host information for those categories, based on our criteria. Taxonomy had the highest number of compliant databases - as 57.9% of them hosted relevant data, based on our criteria. DNA sequences had the lowest number of databases hosting relevant data with a value of 4.14% (Fig. 5). Biodiversity data categories ordered from most to least hosted by databases are: taxonomy (57.9%), geospatial (48.9%), morphology (45.1%), literature (44.4%), temporal data (33.1%), biological collections (21.4%), identification (13.2%), abundance (11.7%) and DNA sequences (4.14%).

## Discussion

### Case-Study

Of the 600 databases we investigated, indexed by TDWG, we found the majority of links unusable, thus making it challenging to address our original questions about types of information in databases and ways this information is structured and accessed in this e-infrastructure. Only 266 of the 600 of them were accessible and contained biodiversity information relevant to our categories (Fig. 2). Our initial reduction of TDWG's index from 600 to 315 databases, suggests a fragile e-infrastructure of the biodiversity database landscape which may be from neglect where the webpage URLs and even the websites themselves had broken links. Furthermore, our observation of the remaining 266 databases indicates that even accessible databases are not necessarily being maintained and updated (Fig. 3). Database disuse appears to have several sources: many are initial start-up projects which fail to remain current once initial funding is exhausted and cannot continue (Canhos et al. 2015, Thomas 2009). The lack of traffic, as a result of little or no publicity, may also contribute to funding and use limitations (Baro et al. 2017). Optimistically, 139 of the 266 databases (52.3%) were currently updated as of 2017, which reaffirms the importance of database management. A negative trend appears between the number of databases and quantity of data categories hosted (Fig. 4). The majority of websites focus on one or two of our categories indicating a pattern of specialisation rather than generalisation. However, the quantity of biodiversity categories a database hosts may not be a direct reflection of the quality of information or its usefulness. This tendency suggests that many databases have relatively specific functions and fill unique niches in the information landscape – suggesting that accessibility of specific data may have a utility that is difficult to translate to mass aggregation.

### Case-Study Limitations

Clearly the results of this study are a small, dated, portion of the current biodiversity information landscape; and we recognise our simplified data categories are hypotheses for revision. Here, we attempt to clarify general issues towards a framework for database classification, to promote this for discussion within the field of biodiversity informatics. We find pervasive accessibility issues, such as link rot, with many databases within the e-infrastructure and we provide evidence that categorising databases, based on the data they contain, is possible. Our defined categories were too few and extremely restrictive – limitations to advance when developing a more mature ontology. Additionally, our scoring of TDWG's list revealed several categories which we did not initially consider. Categories of organism behaviour, traditional knowledge, plant genetic resources, software, proposed standards, credible webinars, environmental DNA (eDNA), legislation and other agreements should be considered. For refining any classification scheme, more granular categories with detailed and precise definitions should be considered. For example, morphological data could have been separated into images of species, precise measurements of physical traits matched to a species specimen and general measurement ranges of morphological traits – indeed, this is the case with some of the smaller, specialist databases. Geospatial data, in particular, could be separated into a multitude of precise categories, based on how data are mapped (points or coloured region) and the resolution of the map itself (country, state, municipalities etc.). These examples underscore the value and importance of a widely-accepted common ontology to categorise biodiversity databases.

### Link Rot

Link rot describes the corruption or removal of hyperlinks and URLs which no longer direct the user to the intended source. Link rot can have many causes and, in this study, we identified three likely circumstances: 1) the intended web page no longer exists; 2) the intended web page has a new domain name; 3) the website hosting the hyperlinks had been restructured, possibly breaking the links. Circumstances 1) and 2) are likely the reason why we could not find 285 of the 600 databases listed on BIPW, because while searching for databases, we often found 404 errors (indicating the page no longer exists) or the domain name was being used for a new website (indicating that the domain had been sold or expired and re-registered). Cause 3) is likely the fate of the BIPW web page, which originally linked to all the databases listed on its web page. However, by the time of this study, every link on the BIPW web page was broken - likely due to a restructuring of the TDWG website.

There are several key ways to prevent link rot. For database curators who are retiring databases or migrating sites to a new domain, they could maintain redirects to the original (or current) source. In addition, for link hosting pages like BIPW, it is important to consistently review and monitor links after local or site-wide updates to repair broken links and flag or follow up resources which do not resolve. However, this may be easier said than done, as the curators of such pages may determine the cost of allocating resources to maintenance outways the usefulness of the database/webpage.

### Ontology Development and Hosting

From observing the databases in this study, we suggest the way forward to provide and promote information about biodiversity databases has two parts: 1) standardised descriptors about a database’s content, organised in an ontology; 2) a stable, yet dynamic, platform for hosting and updating the ontology, with a database index. Standardised descriptors are a foundation of functional databases, because these immediately inform the user about a database’s content, are necessary to aggregate data, can clarify otherwise obscure categories and subcategories and, in the context we propose, facilitates a database’s incorporation into a catalogue. Standardised database coverage descriptors would describe the database itself, the data it hosts and potentially serve as globally unique identifiers – analogous to Digital Object Identifiers (DOI) for publications.

An ontology for categorising biodiversity databases and their content has many best-practice examples to draw from and should incorporate biodiversity scientists from diverse backgrounds to maximise objectivity for developing standard descriptors and structure (FAIRsharing.org 2019, Walls et al. 2014). The resulting ontology should be clearly and publicly available so both the scientific community and general public can understand the ontology structure. As any functional ontology for biodiversity data must evolve, it is vital that the ontology be created to change through regular re-examinations and updates.

We see three possible models for creating a biodiversity database catalogue: 1) the "snapshot"; 2) the “gatekeeper”; and 3) a community-driven method. The snapshot model is a static list that, once curated, does not undergo subsequent revisions or additions; it serves as a snapshot of biodiversity databases at one point in time. In a fast evolving biodiversity e-infrastructure, static lists are quickly outdated and unused. The gatekeeper model is a list curated by one person or a small team that is updated regularly. However, this method is likely to be unsuccessful because of the challenge to keep up with a vast, diverse and ever-growing expanse of databases. It would be incredibly difficult for a small team to survey databases on wide ranges of taxa, geographic regions and time scales to maintain a current and comprehensive list. Obtaining the necessary funding to maintain an effective team could also be another challenge.

Community-driven websites often overcome the limitations intrinsic to the other two models because of the efforts that come from a broad base of curators and contributors. A community-driven approach allows users to collaboratively edit database categories and suggest new categories curated by moderators. This method embraces many individuals from a variety of disciplines and not only allows databases to be categorised at a much faster rate, but also a much wider variety of databases (e.g. different taxa, geographic regions, different languages) can be categorised, as people draw on knowledge from their respective fields. Database creators would also be able to categorise their own databases from intimate knowledge of their databases’s content.

A simple community-driven host for a biodiversity database ontology could work in a similar way to a wiki where users can categorise databases themselves using a pre-defined ontology and then add their categorisation to this common website. Browsed searches on this site could consist of granular categories that will each be appropriately branched into more specific types of databases. For instance, under a general category of databases providing geospatial data, there would be internal categorisation involving range maps provided for continents, range maps at a finer scale with specific regions of a country, range maps with exact GPS points etc. To search for a database that users require, they would select general categories relevant to their research needs, then select subsequent internal categories that apply. Importantly, this method of searching will expose users to databases they may not have been aware of. Consequently, this discovery promotes under-utilised databases to gain publicity and potentially increase their traffic - imparting justification for further funding or development. Community-driven approaches are not without errors, a natural consequence of having many contributors. These problems are, however, often mitigated through peer review and we believe that a community-driven website is the best model to deliver and implement a biodiversity database ontology.

## Conclusions

We developed a simple, but illuminating classification scheme for an indexed list of heterogeneous biodiversity databases, showing how standardised metadata about databases can create a classification ontology for these databases. Such an ontology is necessary for researchers to efficiently find databases containing types of data they need – or discover they need. This ontology should involve database category definitions which are broad, yet detailed enough to provide interoperability for database aggregators and generally increase database accessibility. Categorising biodiversity databases is a daunting task because of the varied information they contain, which may be most feasible when researchers can collaborate through community-driven platforms. Most importantly, our ability to access biodiversity information quickly is imperative for dealing with our global biodiversity crisis. Thus, we hope our modest example in this paper will help to advance conversations about the challenges we face for managing and using biodiversity information.

## Figures and Tables

**Table 1.** A table of the different categories of biodiversity information used to score the biodiversity databases and what each score value means.

	**Score Values**	
**Category**	**0**	**1**
**DNA Sequence**	-has not provided DNA sequences for species or information	-has listed DNA sequences or identifiers for individual organisms
**Identification**	-provided no dichotomous keys	-provided dichotomous keys to assist in identification of species based on physical characteristics
**Abundance**	-does not have quantitative information on the number of individuals	-provides quantitative data on number of organisms in a population or area
**Taxonomy**	-Only listed organism's genus and species -no taxonomic data	-lists taxonomic groups higher than genus
**Literature**	-provided little or no scientific literature	-provided an extensive list of scientific literature (does not have to host the pdf)
**Geospatial**	-no GPS coordinates or plots on a detailed and scaled map of species occurrence	-maps with occurrence plots/points on a map of where individuals were found at a particular point in time or, -provides GPS coordinates
**Biological Collections**	-does not have material sample or entire specimen (living or dead)	-contains material sample(s) or entire (living or dead) specimens
**Morphology**	-provided little or no physical description of organism/species	-provided qualitative data with regards to physical descriptions that are unique to the species or group which aid in identification or, -provided quantitative ranges or exact measurements of an organism's physical features that aid in identification of species
**Temporal**	-no times or dates listed for where an organism was found to occur	- provided a date for when the observation(s) was observed and recorded in terms of where an organism was found
